# Test for Pure Glycerine

**Published:** 1869-01

**Authors:** 


TEST FOR PURE GLYCERINE.—Owing to its impurities, the glycerine of commerce often acts upon the skin and wounds as an irritant, even when diluted with water. The following test serves to distinguish the impure glycerine from that purified by distillation, which possesses such remarkable soothing properties. Mix equal portions of glycerine and rectified sulphuric acid. If the glycerine be pure the mixture will become of a light brown color and will show a certain increase of temperature, and a few bubbles of air will be evolved by shaking. On the contrary, the impure glycerine will give, upon agitation, a large amount of gas. This ceases when the mixture is allowed to remain quiet, but recommences upon a renewal of the agitation.

